# Clonal CD8+ T Lymphocytic Proliferation and Karyotypical Abnormalities in an EBV Associated Hemophagocytic Lymphohistiocytosis

**DOI:** 10.1155/2015/513968

**Published:** 2015-09-16

**Authors:** Jiehao Zhou, Dehua Wang, Mehdi Nassiri

**Affiliations:** ^1^Department of Pathology and Laboratory Medicine, Indiana University, Indianapolis, IN 46202, USA; ^2^Department of Pathology and Laboratory Medicine, Cincinnati Children's Hospital and Medical Center, Cincinnati, OH 45229, USA

## Abstract

EBV associated hemophagocytic lymphohistiocytosis and EBV-positive T cell lymphoproliferative disease of childhood share many histologic and clinical features, which sometimes makes it very difficult to render a definitive diagnosis. In this report, we present a 16-year-old male who developed symptoms clinically consistent with EBV associated hematophagocytic lymphohistiocytosis including fulfilling most of HLH diagnostic criteria and responding promptly to HLH targeted therapy. However, histologic and cytogenetics features of this case are very concerning for EBV-positive T cell lymphoproliferative disease of childhood. This case demonstrates an ambiguous boundary of these two disease entities and emphasizes the importance of comprehensive evaluation and clinical correlation with cases suspicious of EBV driven hemophagocytic or lymphoproliferative process.

## 1. Introduction

Hemophagocytic lymphohistiocytosis (HLH) is clinical syndrome characterized by fever, splenomegaly, pancytopenia, liver dysfunction, and coagulopathy. It is believed that there is an uncontrolled hyperinflammatory process associated with proliferation of activated lymphocytes and histiocytes which secrete excessive amount of proinflammatory cytokines. Histologically, proliferation of lymphocytes, histiocytes, and often hemophagocytosis are seen in bone marrow, spleen, and lymph nodes. There are two forms of HLH: primary HLH and secondary HLH. Primary HLH is an autosomal recessive disease featured by defects in a set of genes including PRF1, UNC13D, or STX11 [1]. Secondary HLH occurs after a strong immunologic activation, such as systemic infection, immunodeficiency, or malignancy. Epstein-Barr virus (EBV) is the most common infectious etiology for development of secondary HLH (EBV-HLH) usually following a primary EBV infection in adolescents. The EBV infests T cells, leading to monoclonal/oligoclonal expansion of T cells. Immunochemotherapy is most common treatment and consists of corticosteroid, etoposide, and cyclosporin [2]. The prognosis is guarded with an overall mortality of ~50%. On the other hand, EBV infection in adolescents can rarely evolve to a malignant T cell proliferative process known as EBV-positive T cell lymphoproliferative disease of childhood as described in WHO hematopoietic disorder classification [3]. This disease is characterized by an extremely high mortality rate despite aggressive management. However, it has significant overlapping features with EBV associated HLH including T cell immunophenotypic aberrancy and T cell clonality, which pose a diagnostic difficulty [4].

Herein, we report a case with clinical features of EBV associated HLH which was associated with a rather abundant clonal CD8+ T cell expansion. These T cells demonstrated aberrant antigen expression and harbored EBV. Cytogenetics study demonstrated abnormal karyotype. These features raised a concern for EBV driven neoplastic process. However, the overall clinical course was more in line with a reactive process.

## 2. Case Presentation

### 2.1. Initial Clinical Presentation

A 16-year-old previously healthy white male initially presented with fever, sore throat, and skin rash involving upper arms and face. A diagnosis of infectious mononucleosis was rendered based on the clinical symptoms and apositive EBV-VCA IgM titer at an outside institute. He was managed by supportive measurements. However, his symptom was worsening and subsequent laboratory studies showed thrombocytopenia with platelets at 56,000 k/*μ*L. Serum transaminases elevated to the ~220 IU/L, and LDH was in the range of 500s IU/L. He was hospitalized and received IV fluids and antibiotics. Nevertheless, his symptoms were further worsening. CBC evaluation showed leukopenia with WBC at 1.5 k/*μ*L and thrombocytopenia with platelets at 36,000 k/*μ*L. In addition, peripheral blood smear showed atypical lymphoid cells. CT study demonstrated hepatosplenomegaly and lymphadenopathy. The patient was transferred to our hospital for further evaluation approximately 6 weeks after initial symptoms.

At the time of transfer, the patient demonstrated profound pancytopenia with white blood cell count at 0.6 k/*μ*L, hemoglobin at 9 g/dL, and platelets at 14 k/*μ*L. Patient also showed renal insufficiency. His creatinine was elevated to 3.2 mg/dL. Additional laboratory studies revealed hyperferritinemia (~48000 *μ*g/L), hypofibrinogenemia (63 mg/dL), markedly elevated soluble IL-2 receptor (~57000 pg/mL), high LDH (2154 IU/L), and mildly increased serum triglyceride. Both EBV-VCA IgM and IgG were positive. PCR study of EBV titer was marked elevated, >7 million copies/mL. Peripheral blood smear showed atypical lymphoid cells with large size, mature chromatin, distinct nucleolus, and basophilic cytoplasm. Molecular genetic testing for familial HLH including SH2D1A, BIRC4, and STXBP2 gene was all normal. The clinical impression was EBV associated secondary hemophagocytic lymphohistiocytosis. A bone marrow evaluation was performed to further characterize the disease.

### 2.2. Pathological, Immunophenotypic, Molecular, and Cytogenetics Findings

**Figure 1:**
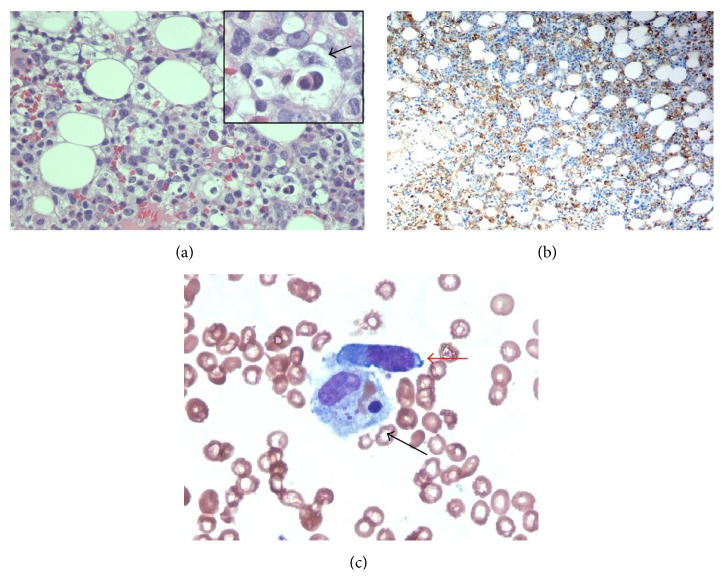
Histologic features of bone marrow. (a) Bone marrow biopsy demonstrated hypocellular marrow with prominent lymphohistiocytic infiltrates. Black arrow in the inset pointed to a histiocyte with engulfed hematopoietic elements. (b) Immunohistochemical stain of CD68 showed increased histiocytes. (c) Bone marrow aspirate smears highlighted hemophagocytosis (black arrow) and atypical lymphoid cell (red arrow).

**Figure 2:**
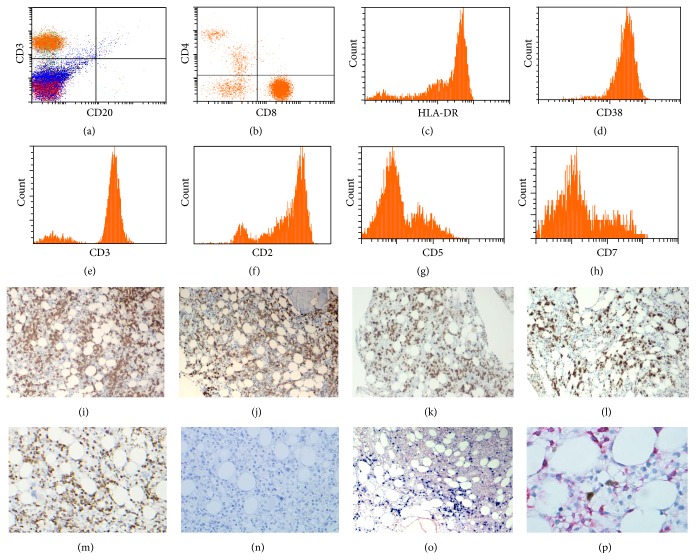
Immunophenotypic features of atypical lymphoid infiltrates. (a)–(h) Flow cytometry analysis of bone marrow showed most of lymphoid infiltrates were CD3+ (a and e), CD2+ (f), CD8+ (b) positive T cells with few CD4+ (b) T cells, and rare CD20+ (a) B cells. These T cells have bright HLA-DR (c) and CD38 (d) expression. Loss of CD5 (g) and CD7 (h) in T cells was evident. (i)–(n) Immunohistochemical stains of bone marrow showed a prominent CD3+ (i) and CD8+ (j) T cell infiltration. These T cells are positive for cytotoxic markers perforin (k), granzyme B (l), and TIA-1 (m) and negative for CD56 (n). (o) In situ hybridization of EBER showed diffuse EBER positive cells in the bone marrow biopsy. (p) Double immunohistochemical stains of CD3 (red) and EBV-LMP (brown) demonstrated colocalization of EBV and CD3.

Bone marrow evaluation showed hypocellular bone marrow with decreased trilineage hematopoiesis and diffuse lymphohistiocytic infiltrates (Figure 1(a)). Increased histiocytes were seen by H&E and immunohistochemical stain of CD68 (Figure 1(b)). Histiocytes with evident phagocytosis were present in both biopsy (Figure 1(a) and inset) and bone marrow aspirate (Figure 1(c)). In addition, there was a very prominent atypical lymphoid cell infiltration composed of lymphoid cells with medium to large size, irregular nuclear contour, and visible nucleolus (Figure 1(a)). Concurrent immunophenotyping by flow cytometric analysis of marrow aspirate also revealed a prominent atypical T cell population which was positive for CD2, CD3, and CD8, with uniformly bright CD38 and HLA-DR expression (Figures 2(a)–2(f)). Loss of CD5 and CD7 antigen expression in these T cells was easily appreciated (Figures 2(g)-2(h)). Immunohistochemical work-up on bone marrow biopsy confirmed that these atypical lymphoid cells are predominant CD3+/CD8+ T cells (Figures 2(i) and 2(j)) with loss of CD5 and CD7 antigen expression. In addition, these atypical lymphoid cells were positive for cytotoxic markers including perforin, granzyme B, and TIA-1 and negative for CD56 (Figures 2(k)–2(n)). These atypical lymphoid cells were also positive for EBV as demonstrated by positive EBV LMP immunohistochemical stain (not shown) and by in situ hybridization of EBER (Figure 2(o)). Double stain of CD3 and EBV LMP demonstrated colocalization of EBV and CD3 (Figure 2(p)).

**Figure 3:**
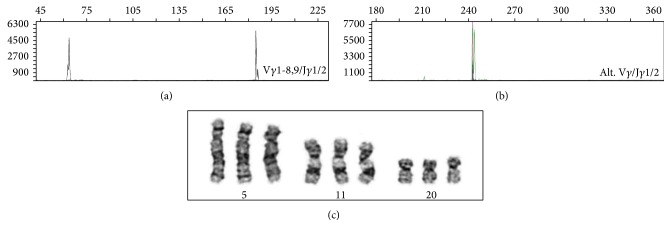
Molecular and cytogenetics findings in the bone marrow. (a) and (b) Assessment of TCR gamma receptor gene rearrangement by PCR on formalin-fixed, paraffin-embedded tissue sections from bone marrow clot demonstrated biclonal PCR product using V*γ* 1-8,9/J*γ*1/2 primer set (a) and monoclonal PCR product using Alt. V*γ*/J*γ*1/2 primer sets (b). (c) G-band karyotypic study on bone marrow aspirate demonstrated an abnormal karyotype in all 20 dividing cells examined: 49,XY,+5,+11,+20/[20].

In order to further characterize this atypical T cell infiltration, PCR of T cell receptor gamma chain gene rearrangement was performed on DNA extracted from bone marrow aspirate. Two clonal TCR-gamma gene PCR products of ~62 and ~185 bp were identified with V*γ* 1-8,9/J*γ*1/2 primer set (Figure 3(a)) and one clonal TCR-gamma PCR product of ~244 bp was identified with Alt. V*γ*/J*γ*1/2 primer sets (Figure 3(b)). Therefore, T cell gene rearrangement study showed a clonal TCR-gamma gene PCR product.

Cytogenetics study on bone marrow aspirate showed an abnormal karyotype on all metaphases examined: 49,XY,+5,+11,+20/[20] (Figure 3(c)).

### 2.3. Treatment and Posttreatment Clinical Findings

Since patient met the proposed diagnostic criteria for HLH, he was started on etoposide, dexamethasone, and Rituxan in addition to supportive care. Cyclosporin administration was initially held due to renal insufficiency. The patient responded to the therapy very well. He was discharged to an outside hospital one week after start of therapy. By the end of 6-week therapy, he was reported to have normal WBC count, mild anemia, mild thrombocytopenia, normal ferritin, fibrinogen, and sIL-2R values. The EBV titer by PCR fell to undetectable level. Posttherapy bone marrow evaluation demonstrated hypocellular bone with trilineage hypoplasia. Neither abnormal lymphoid infiltrates nor overt hemophagocytic activities were identified. Cytogenetic study on the post treatment marrow showed a normal karyotype [46, XY].

The patient was gradually taken off chemotherapy and was followed up clinically. Currently, the patient has been off treatment for three years. He has normal CB except a persistent borderline thrombocytopenia (platelet count is in low 100s). There is no clinical concern of HLH or lymphomatous process.

## 3. Discussion 

Primary infection with the EBV in immunocompetent individuals is characterized by the infection of B lymphocytes by virus via CD21 antigens on the B cells. Usually it is asymptomatic. Some of infected patients develop infectious mononucleosis which is a self-limited disease characterized as strong antibody reaction associated with polyclonal B cell proliferation as well as a robust cytotoxic T cell response which is often monoclonal or oligoclonal [5]. Uncommonly, acute EBV infection leads to the development of hemophagocytic lymphohistiocytosis (HLH), with unregulated activation of histiocytes and often a clonal cytotoxic lymphocyte expansion. Clinical and morphology evaluations demonstrate high fever, hepatosplenomegaly, lymphadenopathy pancytopenia, hemophagocytosis, hypercytokinemia, and multiorgan system failure. Although EBV-HLH is associated with substantial morbidity and mortality, early recognition and prompt immunochemotherapy, such as HLH 2004 protocol, result in successful treatment of EBV-induced HLH. Very rarely, EBV infection results in EBV positive T cell lymphoproliferative disorder of childhood with overt monoclonal T cell proliferation and similar symptoms as those of EBV-HLH. Most of patients with this disease have a fulminant clinical course and extremely high mortality despite aggressive management.

The clinical course and laboratory findings of current case are compatible with EBV associated HLH. For diagnosis of HLH, at least 5 out of 8 findings are required [6]. These findings include (1) persistent fever; (2) splenomegaly; (3) cytopenias in 2 or more cell lines; (4) hypertriglyceridemia (triglyceride level ≥265 mg/dL) or hypofibrinogenemia (fibrinogen level ≤150 mg/dL); (5) hemophagocytosis in the bone marrow, spleen, or lymph nodes; (6) hyperferritinemia (ferritin level ≥500 ng/mL); (7) impaired NK cell function; and (8) elevated soluble IL2 receptor. In our case, seven out of eight findings are identified except for impaired NK cell function which was not performed due to low WBC count in the peripheral blood. Given patient's high EBV titer and negative molecular study result for primary HLH, it is compatible with EBV associated secondary HLH.

In addition to these findings consistent with EBV associated HLH, evaluation of marrow demonstrated a rather abundant abnormal CD3+/CD8+ T cell population which was clonal by TCR gene rearrangement testing. This population was composed of EBV positive and cytotoxic CD8+ T cells with evident aberrant loss of CD5 and CD7 antigen expression. More interestingly, cytogenetic studies of marrow demonstrated a hyperploid karyotype in all cells examined, 49,XY,+5,+11,+20. These findings are highly suspicious for a neoplastic process, particularly systemic EBV-positive T cell lymphoproliferative disorder of childhood as defined in the current WHO classification [3].

Systemic EBV-positive T cell lymphoproliferative disorder of childhood is a rare clonal T cell neoplasm development shortly after primary acute EBV infection in previously healthy children and young adults. Sometimes this entity can arise in a setting of severe chronic active EBV infection. Most of cases were reported in immunocompetent Asians and Native Americans with a very aggressive clinical course and very high mortality. The most classical immunophenotype of this entity is CD3+/CD2+/CD8+ T cells with expression of selective cytotoxic markers including positive for TIA-1 and negative for CD56. Loss of CD5 and CD7 antigen expression in neoplastic T cells is very common. Cases arising in the setting of severe chronic active EBV infection are usually CD4+. Rare cases show neoplastic CD4+/CD8+ double positive T cells. Most of neoplastic cells have clonally rearranged TCR gene and are positive for EBER/EBV-LMP [3].

Although the morphology and immunohistochemical cytogenetics/molecular findings in the bone marrow made us concerned with the diagnosis of EBV-positive T cell lymphoproliferative disease of childhood, our case presented with a few unusual characteristics when compared to the more typical case of EBV-positive T cell lymphoproliferative disorder of childhood. First of all, typical EBV-positive T cell lymphoproliferative disorder of childhood is usually found in children or adolescents of Asian or Native American origin [3]. It is hypothesized that a genetically determined susceptibility leads to an abnormal response to primary EBV infection. By contrast, our patient is a white male without known immunodeficiency. Secondly, a relatively common finding in typical EBV-positive T cell lymphoproliferative disease of childhood is the absence of serologic response to EBV. EBV anti-VCA IgM was negative in the majority of reported typical cases whereas EBV anti-VCA IgG is either absent or only moderately elevated [7]. Sometimes, the negative EBV serology result may be clinically misleading since it does not indicate an ongoing EBV infection, which may lead to a wrong diagnosis path. It has been speculated that the lack of humoral response to EBV results in virus proliferation in T cells and subsequently development of clonal expansion of EBV positive T cells. In contrast, our patient demonstrated a relatively robust antibody response to EBV with both positive EBV anti-VCA IgG and IgM (titer up to 1 : 1280). Ultimately, systemic EBV-positive T cell lymphoproliferative disorder of childhood has a very aggressive clinical course with extremely high mortality which is thought to be related with uncontrolled HLH. Most of cases have a fulminant course resulting in death in days to month. Only extremely rare cases showed clinical remission after aggressive chemotherapy. Our patient had a very good response to HLH 2004 protocol treatment. Liver function, hematology index, and viral titers all recovered following treatment. Bone marrow examination ~2 months after treatment showed no evidence of clonal T cell infiltration and normal cytogenetics. Overall these findings are more compatible with self-limited clonal T cell proliferation associated with EBV associated HLH.

Aberrant antigen expression in T cells and monoclonal and oligoclonal T cell expansion in infectious mononucleosis and EBV associated HLH has been reported previously. Weisberger et al. [8] demonstrated that acute infectious mononucleosis is featured by expansion of activated CD8+ cytotoxic T cells with frequent downregulation of CD7 antigen. Bright CD38 and HLA-DR antigen expression in T cells are noted. Not uncommonly, molecular studies demonstrated clonal T cell population. Weisberger et al. [8] had proposed that the bright expression of activation marker of HLA-DR served as a clue of reactive nature as most of peripheral T cell neoplasms often have absent to heterogenous expression of HLA-DR. On the other hand, Toga et al. [9] reported expanded CD8+ cytotoxic T cells in peripheral blood of six EBV associated HLH pediatric patients in Japan. Different from T cells in infectious mononucleosis, these T cells demonstrated more evident downregulation of CD5 antigen in addition to loss of CD7 and bright HLA-DR expression by immunophenotyping. Flow cytometry analysis of TCR V*β* and PCR analysis of TCR beta gene rearrangement analysis demonstrated T cell clonality in the majority of patients. Moreover, immunohistochemical studies revealed that these CD8+ T cells were cellular target of EBV infection as compared to the B cells which were target of EBV infection in infectious mononucleosis. Toga proposed that clonal expansion of CD8+ T cells with CD5 downregulation as characteristic immunophenotypic features of EBV associated HLH as a tool to distinguish patients with infectious mononucleosis since no such population was identified in patients with infectious mononucleosis.

Cytogenetics abnormalities have been reported sporadically in EBV associated HLH [10–12]. Imashuku et al. [11] showed that the EBV associated HLH with abnormal karyotype is comprised of ~20% case in a retrospective cohort of 32 EBV associated HLH cases and was invariably fatal with a 3-year survival of 14%. The majority of cytogenetically abnormal clone demonstrated hyperdiploid. However, no recurrent clonal chromosomal abnormality has been documented in HLH cases with cytogenetics abnormalities in their series [11]. A separate study led by Chen et al. [12] showed 3 cases of EBV-HLH with cytogenetics abnormality at a single medical center. There is a recurrent karyotypic abnormality of add(9)(p24) in two of them. Our patient demonstrated a nonrandom hyperploid karyotype 49, XY,+5,+11,+20 in all 20 metaphases examined. Usually, clonal karyotypic abnormality in this clinical setting is an indication of neoplastic process. However, our patient recovered rather promptly after typical courses of immunochemotherapy for HLH. Posttreatment study demonstrates normal karyotype and the long term follow-up (3-year) showed no evidence of reoccurrence of disease.

In summary, we report a rare case of EBV-HLH associated with evident clonal CD8+ T cell expansion. The abundance of T cell infiltration in the bone marrow, aberrant immunophenotypic features, clonality by TCR gamma gene rearrangement, and abnormality by karyocytic cytogenetics study are highly suspicious for EBV-positive T cell lymphoproliferative disorder of childhood. However, the clinical course and responsiveness to the immune chemotherapy are more in line with a reactive process. This case further highlights the diagnostic difficulties to distinguish EBV driven reactive versus malignant process. Comprehensive evaluation including morphological, immunophenotypic, molecular, and karyotypical studies is important. Clinical correlation, prompt treatment, and posttreatment follow-up are essential.

## References

[1] Henter J.-I., Arico M., Elinder G., Imashuku S., Janka G. (1998). Familial hemophagocytic lymphohistiocytosis. Primary hemophagocytic lymphohistiocytosis. *Hematology/Oncology Clinics of North America*.

[2] Imashuku S. (2011). Treatment of Epstein-Barr virus-related hemophagocytic lymphohistiocytosis (EBV-HLH); update 2010. *Journal of Pediatric Hematology/Oncology*.

[3] Quintanilla-Martinez L., Kimura H., Jaffe E. S., Swerdlow S. H., Campo E., Harris N. L. (2008). EBV-positive T-cell lymphoproliferative disorders of childhood. *WHO Classification of Tumours of Haematopoietic and Lymphoid Tissues*.

[4] Smith M. C., Cohen D. N., Greig B. (2014). The ambiguous boundary between EBV-related hemophagocytic lymphohistiocytosis and systemic EBV-driven T cell lymphoproliferative disorder. *International Journal of Clinical and Experimental Pathology*.

[5] Callan M. F. C., Steven N., Krausa P. (1996). Large clonal expansions of CD8^+^ T cells in acute infectious mononucleosis. *Nature Medicine*.

[6] Henter J.-I., Horne A., Aricó M. (2007). HLH-2004: diagnostic and therapeutic guidelines for hemophagocytic lymphohistiocytosis. *Pediatric Blood and Cancer*.

[7] Quintanilla-Martinez L., Kumar S., Fend F. (2000). Fulminant EBV^+^ T-cell lymphoproliferative disorder following acute/chronic EBV infection: a distinct clinicopathologic syndrome. *Blood*.

[8] Weisberger J., Cornfield D., Gorczyca W., Liu Z. (2003). Down-regulation of pan-T-cell antigens, particularly CD7, in acute infectious mononucleosis. *American Journal of Clinical Pathology*.

[9] Toga A., Wada T., Sakakibara Y. (2010). Clinical significance of cloned expansion and CD5 down-regulation in epstein-barr virus (EBV) infected CD8^+^ T lymphocytes in EBV-Associated hemophagocytic lymphohistiocytosis. *Journal of Infectious Diseases*.

[10] Kaneko Y., Maseki N., Sakurai M. (1995). Clonal and non-clonal karyotypically abnormal cells in haemophagocytic lymphohistiocytosis. *British Journal of Haematology*.

[11] Imashuku S., Hibi S., Tabata Y. (2000). Outcome of clonal hemophagocytic lymphohistiocytosis: analysis of 32 cases. *Leukemia & Lymphoma*.

[12] Chen J.-S., Tzeng C.-C., Tsao C.-J. (1997). Clonal karyotype abnormalities in EBV-associated hemophagocytic syndrome. *Haematologica*.

